# Coping with Unpredictability: Dopaminergic and Neurotrophic Responses to Omission of Expected Reward in Atlantic Salmon (*Salmo salar* L.)

**DOI:** 10.1371/journal.pone.0085543

**Published:** 2014-01-17

**Authors:** Marco A. Vindas, Christina Sørensen, Ida B. Johansen, Ole Folkedal, Erik Höglund, Uniza W. Khan, Lars H. Stien, Tore S. Kristiansen, Bjarne O. Braastad, Øyvind Øverli

**Affiliations:** 1 Department of Animal and Aquacultural Sciences, Norwegian University of Life Sciences, Ås, Norway; 2 Department of Biosciences, University of Oslo, Oslo, Norway; 3 Department of Animal Welfare, Institute of Marine Research, Matredal, Norway; 4 Department of Marine Ecology and Aquaculture, Danish Institute for Fisheries Research, Hirtshals, Denmark; Medical University of South Carolina, United States of America

## Abstract

Comparative studies are imperative for understanding the evolution of adaptive neurobiological processes such as neural plasticity, cognition, and emotion. Previously we have reported that prolonged omission of expected rewards (OER, or ‘frustrative nonreward’) causes increased aggression in Atlantic salmon (*Salmo salar*). Here we report changes in brain monoaminergic activity and relative abundance of brain derived neurotrophic factor (BDNF) and dopamine receptor mRNA transcripts in the same paradigm. Groups of fish were initially conditioned to associate a flashing light with feeding. Subsequently, the expected food reward was delayed for 30 minutes during two out of three meals per day in the OER treatment, while the previously established routine was maintained in control groups. After 8 days there was no effect of OER on baseline brain stem serotonin (5-HT) or dopamine (DA) activity. Subsequent exposure to acute confinement stress led to increased plasma cortisol and elevated turnover of brain stem DA and 5-HT in all animals. The DA response was potentiated and DA receptor 1 (D1) mRNA abundance was reduced in the OER-exposed fish, indicating a sensitization of the DA system. In addition OER suppressed abundance of BDNF in the telencephalon of non-stressed fish. Regardless of OER treatment, a strong positive correlation between BDNF and D1 mRNA abundance was seen in non-stressed fish. This correlation was disrupted by acute stress, and replaced by a negative correlation between BDNF abundance and plasma cortisol concentration. These observations indicate a conserved link between DA, neurotrophin regulation, and corticosteroid-signaling pathways. The results also emphasize how fish models can be important tools in the study of neural plasticity and responsiveness to environmental unpredictability.

## Introduction

The evolution of complex neural phenomena such as cognition and emotion has received increasing scientific attention in the last few decades. Comparative studies on teleost fish models have questioned whether the existence of cognitive abilities (i.e. attention, perception, memory formation) in these animals render them capable of consciously experiencing affective states such as stress, pain and frustration. Furthermore, it is of interest to ascertain to which degree such cognitive capacities merit concern related to animal welfare [1]–[3]. Although the bulk of knowledge on these subjects stems from mammalian neuroscience research, it is believed that emotional responses are evolutionarily adaptive (e.g. inducing appropriate behavioral responses to dangerous and rewarding stimuli). It is thus plausible that the same principles apply in fishes and mammals [4]–[6]. Vital to forming appropriate behavioral responses is behavioral plasticity; the ability to respond differently to the same stimulus depending on experience, sensory information and internal state [7], [8]. In fishes as well as in mammals, the biological basis of behavioral plasticity is neural plasticity [8]–[11], which can be categorized into two main forms: *Biochemical switching*, including modulation of the neural output to a stimulus by neuromodulators such as monoamine neurotransmitters; and *structural reorganization* of neural networks including long-term potentiation (LTP), neurogenesis and dendritic arborization [7], [8], [10].

In terrestrial vertebrates, unforeseen omission of expected reward (OER) typically elicits an emotional response termed frustration [12]. Amsel [13] defined frustration as “an aversive motivational state preceded by the omission of an expected reward”. In humans, frustration following OER leads to activation of brain areas associated with physical and emotional pain [14]. Such a motivational state is likely to affect behavior, and indeed, OER-induced frustration induces aggression in both mammals and birds [15]–[18]. Recently we reported that OER also increases aggressive behavior in a teleost model, the Atlantic salmon (*Salmo salar*) [19].

In mammals, OER also increases levels of attention and learning [20], [21], although the molecular mechanisms are still unclear. In fishes, the role of monoamine neurotransmission in behavior has been extensively studied in diverse behavioral contexts, such as stress and social interaction (for reviews see Winberg and Nilsson [22] and Maximino and Herculano [23]), and both serotonin (5-hydroxytryptamine, 5-HT) and dopamine (DA) are involved in regulation of aggression and social status [22], [24], [25]. In mammals, strong links between the DA system and both aggression and reward signaling are well established [21], [26], [27]. 5-HT has also recently been shown to be involved in reward learning in rats [28]. Therefore, both DA and 5-HT are candidates for mediating the reported behavioral effects of OER in Atlantic salmon. Monoamine neurotransmitters are also potent regulators of structural plasticity, including neurogenesis and LTP [29]–[31], and potential long-term effects of a behavioral paradigm like OER are likely to be embedded in neural circuits through structural plasticity. Recent studies have reported changes in markers for structural plasticity in the fish brain in response to a range of factors including acute stress, chronic stress and environmental enrichment [10], [32]–[35]. One such marker is proliferating cell nuclear antigen (PCNA), which is expressed in actively cycling progenitor cells, and thus reflects cell proliferation [10], [32], [35]–[38]. Another, brain-derived neurotrophic factor (BDNF), is vital for several aspects of structural neural plasticity in mammals [39]. BDNF has also recently been linked with reward-associated learning in mice [40].

Thus, we hypothesize that unpredictable omission of expected reward will induce changes in neural plasticity in Atlantic salmon that are suggestive of mild chronic stress, and that this in turn, will alter responsiveness to additional novel stressors. Therefore, in both undisturbed and acutely stressed salmon from the previously established OER paradigm [19], we quantify the neural plasticity marker abundance of BDNF and PCNA in the telencephalon, an area important for control of social interaction and aggression [41]. Furthermore, we quantify the abundance of DA receptors D1 and D2, which are involved in reward association in mammals [21], [27]. In addition, we report neurochemical indices of DA and 5-HT activity in the brain stem, which, like in mammals, contains important monoaminergic nuclei innervating large parts of the brain [42], [43].

## Materials and Methods

### Ethics statement

This work was conducted in accordance with the laws and regulations controlling experiments and procedures on live animals in Norway and was approved by the Norwegian Animal Research Authority (NARA), following the Norwegian Regulation on Animal Experimentation Act of 1996. All fish were monitored for injuries throughout the experiment. Sampling procedures are described in section 2.5.

### Animals and experimental set-up

The experiment was conducted at the Institute of Marine Research, Matre Research Station in November and December 2010. 1200 Atlantic salmon smolts (commercial strain, Aquagen AS) were transferred from outdoor rearing tanks and randomly distributed in 6 indoor circular tanks (diameter  = 3 m, water depth  = 0.75 m, volume  = 5.3 m^3^). After 12 days, all fish (n = 200 per tank) were anesthetized using MS-222 (Finquel®, Argent Chemical Laboratories, Redmond, WA, USA) at a concentration of 0.1 g/L for weighing, measurement and tagging. The fish had a mean fork length of 21.0±1.2 cm and weight of 103±21 g (mean ± SD). All individuals were also marked with numbered T-bar anchor tags (2 cm long, Hallprint, Pty Ltd. Australia) attached behind the dorsal fin. Fish were then left to recover for a period of 15 days, during which they were monitored for infections resulting from the tagging procedure. The tanks were kept under a simulated natural light regime, with sunrise and sunset progressing from 08:04 h/16:41 h to 08:44 h/16:04 h throughout the course of the experiment. Light was supplied by fluorescent light tubes (Philips, TL-D 36W/33-640) positioned centrally above each tank. The saltwater flow (salinity of 34.3 ppt) was kept at 140 L/min, maintaining oxygen saturation between 75 and 90% in the outlet water. The water temperature was kept at 16°C. A computer system connected to a feeder (Arvotec feeding units: Arvo-Tec T drum 2000, www.arvotec.fi, Finland) controlled food amount and delivery times. The food consisted of 4 mm dry pellets (Skretting AS, Norway).

The fish (n = 200 per tank) were conditioned for 22 days using a delay conditioning regime with a flashing light as the conditioned stimulus (CS) and feeding as the unconditioned stimulus (US) (Vindas et al. 2012). The flashing light (1 s on and 2 s off) was delivered via a light bulb (12 V, 21 W) positioned 10 cm above the food entrance point (light intensity was 5.4 mE, Li-Cor spqa 193A spherical sensor). The CS began 30 s before the onset of US and overlapped 10 s with the US, i.e. creating a delay-conditioning regime [44]. Programmable relays (Ocean Controls, KT-5074APC Printer Port Relay Board Assembled, Australia) activated both the CS and US. The percentage of food (i.e. % per total fish body mass per tank) was slowly decreased throughout the course of the conditioning period in order to increase feeding motivation. During the first 15 days of conditioning, the fish were fed 7 equally sized meals, for 5 min hourly from 09:00 h to 15:00 h, in total equivalent to 2% of their body mass per day. On day 16, the food ration was decreased to 1.5% of calculated body mass for all fish. Finally, 2 days before the start of OER, food was presented in all tanks 3 times a day at 09:00 h, 12:00 h and 15:00 h over a period of 10 min, and the ration was lowered again, this time to 1.25% of body mass. This was done to further sensitize individuals to the CS, as when hunger levels rise, the motivation for foraging and foraging related activities (e.g. learning about food) is believed to surpass other pressures, such as mating, antipredator behavior and willingness to incur/avoid social interaction that may lead to social contests [45]. Fish were weighed before tagging at the start of the experiment and at sampling in order to calculate specific growth rates (SGR: % increase body w/d). One diseased fish was excluded before the start of OER.

### Omission of expected reward

After conditioning, OER was conducted in 3 of the tanks over 9 days, while the remaining 3 tanks serving as control groups were kept on identical feeding patterns, without OER. For OER, the food reward was delayed by 30 min after initiation of the CS. This was done during the first two meals of each day, at 9:00 h and 12:00 h, but not during the last meal at 15:00 h, when all tanks were again treated equally (i.e. food was presented 30 s after initiation of the CS). This variability was introduced partly in order to increase unpredictability in presentation of the food reward, and partly to retain the associative value of the CS to the US.

### Behavioral analysis

Behavioral data from the current study was published in Vindas et al. [19]. For clarity, the methods will be briefly described here. Video recordings were used to monitor behavioral responses in the CS/US area (i.e. the area immediately under the light signal/feeder, representing a quarter of the total size of the tank). During the conditioning period the system was programmed to record twice during each meal at: (i) 1 min from 30 s before onset of the CS to 30 s after onset of the CS, and (ii) 1 min during US, starting 4 min after the end of the CS. During OER, recording was started 5 min before onset of the CS and ended 15 min after the end of the CS (approximately 15 min before onset of the delayed US). To establish the response to the conditioning regime, image frames were captured from videos at 10 s before and 20 s after onset of the CS (before food delivery). We quantified the change in number of individuals in each tank (n = 200) present in this area in response to the CS. In order to measure aggression (aggressive acts/min), the whole tank area was analyzed for total observable aggressive acts, i.e. charges, nips and chases [47]. This was done for a total of 5 min at 2 different time points: from 6 to 1 minute before CS and from 10 to 15 min after CS for all three daily meals.

### Sampling, confinement test and brain dissection

After 8 days of OER, four randomly chosen fish per tank (12 per treatment group) were collected by use of a dip net 45–60 min after the second CS (i.e. 15–20 min after OER fish had received their delayed food reward). On the following day, before the first meal, 6 randomly chosen fish per tank were similarly collected in order to be subjected to an acute confinement stress by individually placing them in perforated see-through plastic containers (26 cm×15 cm×6 cm) submerged in water for a period of 30 min [46]. For sampling purposes all fish were deeply anesthetized with MS222 (0.1 g/L) until there was no observable opercular movement (approximately 10–15 s), either immediately after collection from the tank (unstressed conditions) or following individual confinement stress. Fish were measured and weighed, yielding 227.5±7 g and 25.9±0.2 cm in control, and 219.1±9.7 g and 25.9±0.6 cm in OER fish, before a blood sample was taken from the base of the caudal fin using 1 ml injections containing the anticoagulant ethylene di-amine tetra acetic acid (EDTA). The blood sample was centrifuged for 5 minutes at 9280 rcf and 4°C, and the supernatant was frozen and stored at −80°C for later analysis. The fish were decapitated and brains were quickly excised (within 2 min) to sample the telencephalon (excluding olfactory bulbs) and the brain stem (excluding the cerebellum). Brain stems were wrapped in individually marked aluminum foil packets, snap-frozen in liquid nitrogen and stored at −80°C for later monoamine analysis. Telencephalon samples were kept in 1.5 ml Eppendorf tubes containing RNA later® (Ambion, Austin, TX, USA) for 12 hours at 2°C before storage at −80°C for later mRNA analysis.

### Brain monoamine neurochemistry

Frozen brain stems were homogenized in 4% (w/v) ice-cold perchloric acid (PCA) containing 0.2% EDTA and 40 ng/ml epinine (deoxyepinephrine as an internal standard) with a Potter–Elvehjem homogenizer. After centrifuging samples for 5 min at 15493 rcf, the supernatant was analyzed by high-performance liquid chromatography (HPLC). The mobile phase was made up of 12 µmol/L EDTA, 86 mmol/L sodium phosphate and 1.4 mmol/L sodium octyl sulphate in deionized water (resistance 18.2 MW), containing 7% acetonitrile brought to pH 3.1 with phosphoric acid. The system used a solvent delivery system (Shimadzu, LC-10AD), an auto-injector (Famos, Spark), a reverse phase column (4.6 mm*100 mm, Hichrom, C18, 3.5 mm) and an ESA Coulochem II detector (ESA, Bedford, MA, USA) with two electrodes at −40 mV and +320 mV. A conditioning electrode with a potential of +40 mV was used to oxidize possible contaminants before analysis. Brain stem concentrations of DA, the DA metabolite 3,4-Dihydroxyphenylacetic acid (DOPAC), 5-HT and the 5-HT metabolite 5-Hydroxyindoleacetic acid (5-HIAA) were quantified by comparison with standard solutions of known concentrations and corrected for recovery of the internal standard using HPLC software (CSW, DataApex Ltd, the Czech Republic).

### Gene mRNA abundance

Total RNA was extracted from the telencephalon using lipid-tissue RNA isolation mini kit (QIAGEN, West Sussex, UK). RNA was treated with Turbo DNA-free™ (Ambion, Austin, TX, USA) to avoid genomic contamination before cDNA synthesis. A poly dT-primer and SuperScript™ III Reverse Transcriptase (Invitrogen, Carlsbad, CA, USA) were used to synthesize cDNA. RNA (200 ng/ml to a total of 5 µl) was added to each reaction, to a total reaction volume of 20 µl. Partial sequences for D1 (accession # EU371401.1), BDNF (accession #GU108576.1) and PCNA (accession # BT056931) were retrieved from the National Center for Biotechnology Information (NCBI, http://www.ncbi.nlm.nih.gov/nuccore) and the sequence for D2 (accession # GE767991.1) was retrieved from the gene index project (DCFI, http://compbio.dfci.harvard.edu/temp/blastn-salmon-21392-1373357525.html). Primers were synthetized using the program Primer3 (http://frodo.wi.mit.edu/primer3/input.htm) and synthetized by Invitrogen. A minimum of 4 different primer pairs were designed at exon junctions for each gene, and for each gene, the primer pair showing the lowest crossing point value (Cp) and a single-peak melting curve was chosen. The PCR products were sequenced to confirm the desired primer cDNA amplification. A previously established housekeeping gene, EF1α [48] was used as an internal control gene, as its abundance was stable between experimental groups and did not display any treatment effects (data not shown).

The samples were analyzed by quantitative real-time reverse transcriptase PCR (qtRT-PCR) following the procedure described by Johansen et al. [49]. In short, qtRT-PCR was carried out using a Roche LC480 light cycler (Roche Diagnostics, Penzberg, Germany). Reaction volume was 10 µl, including Light cycler® 480 SYBR Green I Master (Roche diagnostics GmbH, Mannheim, Germany), primers (5 µM each) and cDNA. Cycling conditions were as follows: 10 min at 95°C, 42 cycles of 10 s at 95°C, 10 s at 60°C and 10 s at 72°C followed by melting curve analysis. All reactions were run in duplicates and controls without DNA templates were run to verify the absence of cDNA contamination. Relative gene abundance data was calculated from qtRT-PCR raw data using formula (1). 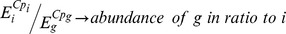
(1)where I is internal control gene (EF1α), G is gene of interest, E is priming efficiency, and Cp is crossing point value. E values were calculated for each qtRT-PCR reaction using LinRegPCR software (version 11.30.0) [50].

### Radioimmunoassay for cortisol

Plasma samples were diluted in 1∶2 RIA buffer (containing 0.05% NaN_3_), followed by heat-treatment for 1 h at 80°C to denature proteins. After cooling for 1 h, samples were centrifuged at 1384 rcf for 20 min at 4°C, after which the supernatant containing cortisol was collected and stored at 4°C. Samples were assayed in duplicate, with all tubes containing 100 µl of plasma sample, 200 µl of anti-cortisol antibody (Abcam, ab1949) and 50 µl of hydrocortisone (1, 2, 6, 7-^3^H (N), Perkin Elmer). Plasma concentration of cortisol was measured by specific radioimmunoassay (RIA) following the procedure described by Mayer et al. [51], which includes a comprehensive validation of the steroid RIA for Atlantic salmon plasma, with comparisons between the above heat-treatment method with extraction followed by thin-layer chromatographic (TLC) separation of the steroids. As there was no significant difference between the two methods, the heat-treatment method was chosen. The intra- and inter-assay coefficients of variance for the cortisol assay were 6.3% and 12.1%, respectively.

### Statistical analysis

Homogeneity of variance was checked by Levene's test and log or arcsine transformation was used to achieve homogeneity when required. One-way ANOVA was used to confirm a significant response to the conditioning regime (a detailed analysis of the conditioned response is presented in [19]). For the neuroendocrine variables (brain stem DA and 5-HT activity, telencephalic D1, BDNF and PCNA mRNA abundance, and plasma cortisol levels) a one-way ANOVA comparing 4 groups (i.e. unstressed controls, stressed controls, unstressed OER and stressed OER) was conducted. This model used group as a fixed effect variable and individual data nested within rearing unit as a random effect variable. Significant group variation was further analyzed by a *Tukey Honest Significant Differences* (HSD) post-hoc test. In one case, for D1 abundance, the ANOVA indicated a significant effect but no post-hoc significance was identified. Consequently, an additional model was tested with treatment (OER vs. control) as the fixed effect. Relative D2 abundance did not achieve variance homogeneity by transformation, so in this case a non-parametric one-way *Kruskal-Wallis* test was used. *Spearman's* correlation analysis (on non-transformed data) was used to analyze the relationship between the significantly affected neuroplasticity indicator, BDNF mRNA abundance, and two other variables: D1 receptor mRNA abundance and plasma cortisol concentrations.

## Results

### Behavior, growth rates and plasma cortisol

**Figure 1:**
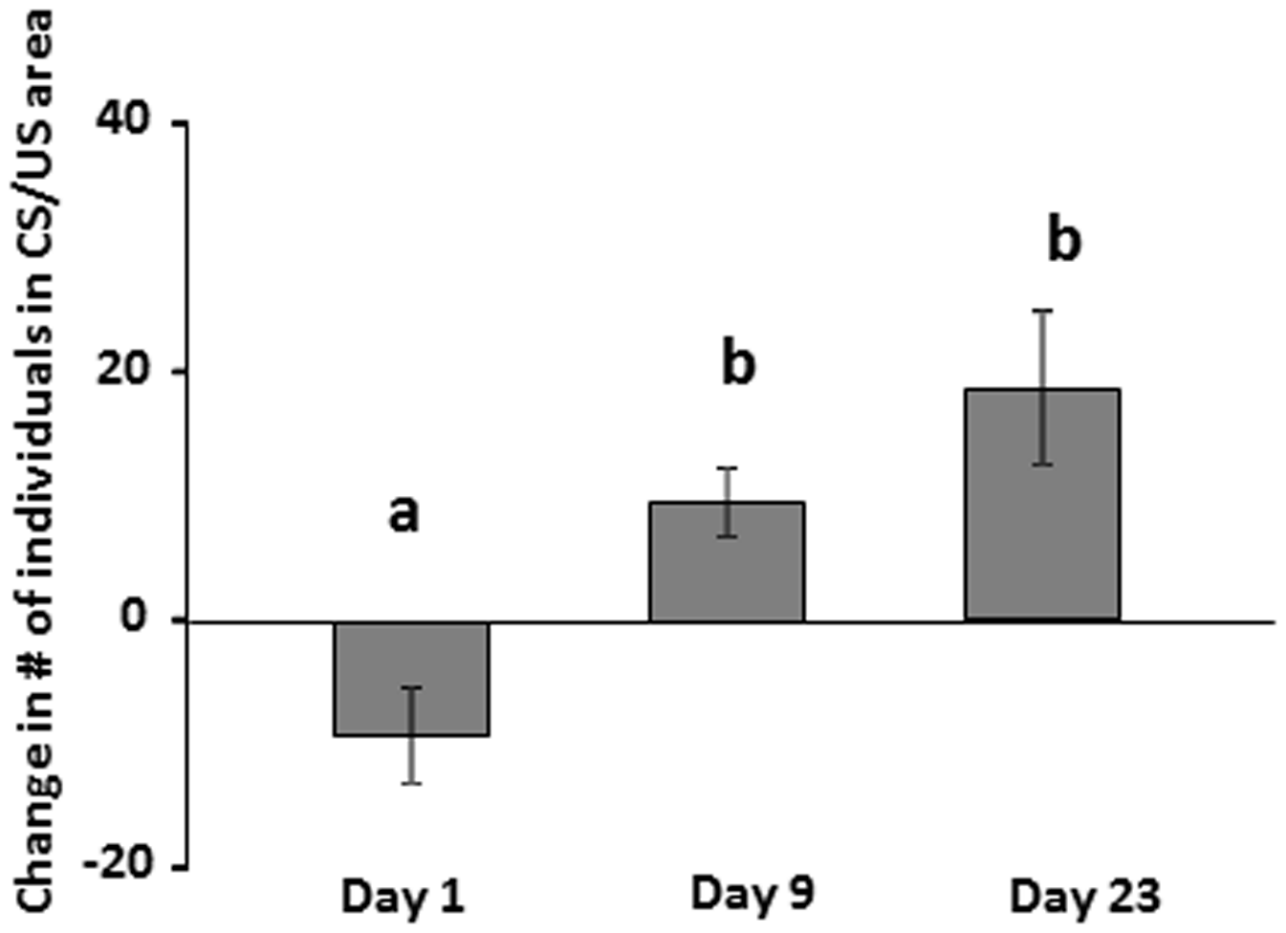
Magnitude of the conditioned response depicted as the change in number of individuals present in CS/US area (number of fish during signal minus number of fish before signal), during the conditioning period. *ANOVA*: *p* = 0.002. *Tukey HSD* post-hoc significance levels are indicated by differing letters when *p*<0.05 (The figure has been constructed from data previously reported in Vindas et al. [19]).

An initial avoidance response to the CS was observed upon exposure to the conditioning regime, but the fish quickly started accumulating in the CS/US area in response to expected food delivery (Figure 1, and see [19] for detailed time-course data). Furthermore, the OER treatment resulted in a 51% increase in observable aggressive acts immediately after the CS (see [19] for details and statistics). Growth rates were not statistically different between treatments, but OER caused a 53% increase in growth variability compared to control groups [19].

Plasma cortisol levels were not significantly different between control fish (Unstressed: 9.0±0.7; Stressed: 55.6±5.4 ng/ml) and OER fish (Unstressed: 9.1±0.5; Stressed: 65.1±4.5 ng/ml) neither under basal conditions nor after acute stress. However, both control and OER groups reacted with significantly increased cortisol levels in response to acute restraint stress (see Vindas et al.[19] for details and discussion of these results).

### Monoamine neurochemistry

**Figure 2:**
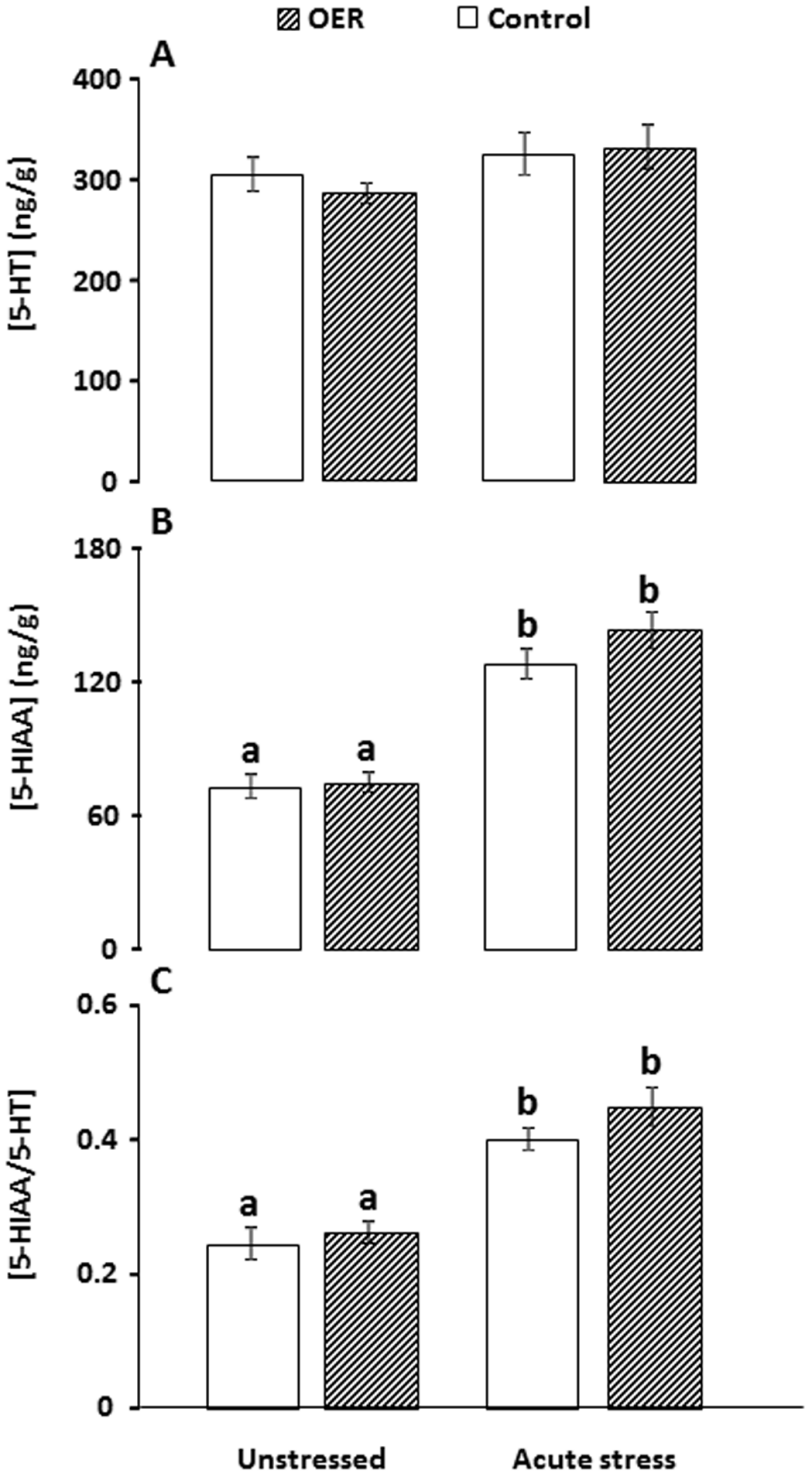
Mean concentrations (ng/g) of A) Serotonin (5-HT), B) 5-HIAA and C) the 5-HIAA/5-HT ratio in brain stem of omission of expected reward (OER) and control groups under unstressed and stressed conditions. *ANOVA* statistics, A: *F*_(3)_ = 1.07, *p* = 0.37, B: *F*_(3)_ = 27.45, *p*<0.001, C: *F*_(3)_ = 17.89, *P*<0.001. *Tukey HSD* post-hoc significance levels are indicated by differing letters when *p*<0.05.

**Figure 3:**
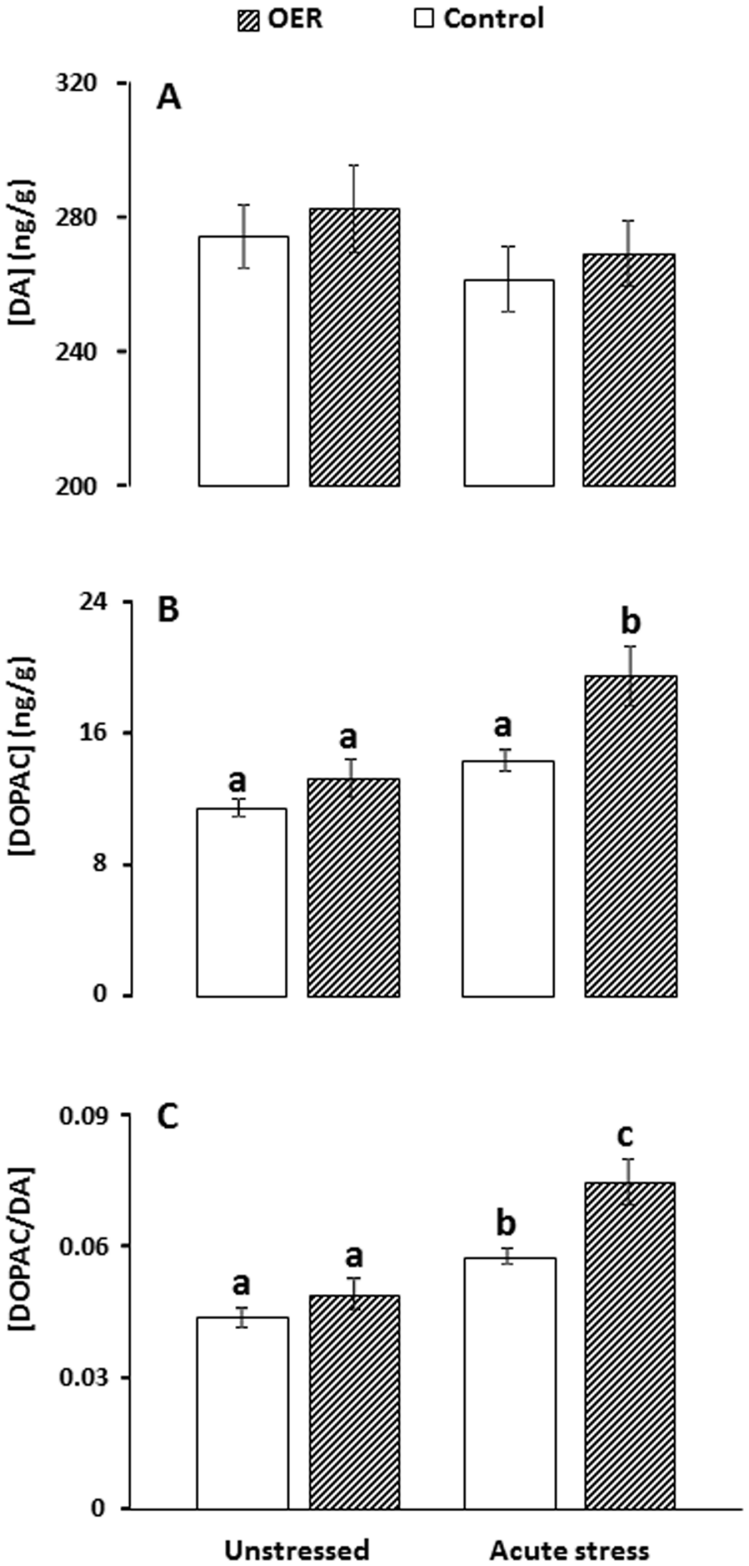
Mean concentrations (ng/g) of A) Dopamine (DA), B) DOPAC and C) the DOPAC/DA ratio in brain stem of omission of expected reward (OER) treated and control groups under unstressed and stressed conditions. *ANOVA* statistics, A: *F*_(3)_ = 0.69, *p*<0.56, B: *F*_(3)_ = 10.55, *p*<0.001, C: *F*_(3)_ = 15.81, *p*<0.001. *Tukey HSD* post-hoc significance levels are indicated by differing letters when *p*<0.05.

Results of neurochemical measurements are presented in Figure 2 (5-HT) and Figure 3 (DA). There were no group differences in brain stem 5-HT concentrations (*p* = 0.3, see detailed ANOVA statistics in caption of figure 2). Indicators of 5-HT activity, i.e. 5-HIAA concentrations and 5-HIAA/5-HT ratio were not significantly different between OER and control groups under unstressed conditions or after acute stress (see Table1 for specific post-hoc *p* values associated with each group comparison). However, both control and OER treated fish reacted with increased 5-HT activity after acute stress. Brain stem DA concentration was not significantly different between groups (*p* = 0.56, figure 3). DOPAC concentrations and the DOPAC/DA ratio were not significantly different between treatment groups under unstressed conditions. Both treatment groups responded with increased DOPAC/DA ratios to acute confinement stress, while only OER fish had significantly increased DOPAC levels after confinement stress. Furthermore, OER animals showed a more pronounced DA response to confinement stress, compared to control groups (Figure 2 and Table 1).

**Table 1:** *ANOVA* statistics and *Tukey HSD* post-hoc test for brain serotonin (5-HT), 5-HIAA, 5-HIAA/5-HT, dopamine (DA), DOPAC, DOPAC/DA, dopamine receptors 1 (D1) and 2 (D2), brain derived neurotrophic factor (BDNF) and proliferating cell nuclear antigen (PCNA) of omission of expected reward (OER) treated and control fish under unstressed and acute stress conditions.

		Treatment effect		Effect of stress	
	ANOVA	Unstressed	Stressed	Control	OER
5-HT	*F*_(3)_ = 1.07, *p* = 0.37	—	—	—	—
5-HIAA	*F*_(3)_ = 27.45, *p*<0.001	*p* = 0.99	*p* = 0.32	***p*** **<0.001**	***p*** **<0.001**
5-HIAA/5-HT	*F*_(3)_ = 17.89, *p*<0.001	*p* = 0.97	*p* = 0.42	***p*** **<0.001**	***p*** **<0.001**
DA	*F*_(3)_ = 0.69, *p*<0.56	—	—	—	—
DOPAC	*F*_(3)_ = 10.55, *p*<0.001	*p* = 0.57	***p*** ** = 0.01**	*p* = 0.08	***p*** ** = 0.001**
DOPAC/DA	*F*_(3)_ = 15.81, *p*<0.001	*p* = 0.7	***p*** ** = 0.005**	***p*** ** = 0.01**	***p*** **<0.001**
D1	*F*_3_ = 3.14, *p* = 0.03	*p* = 0.09	*p* = 0.23	*p* = 0.99	*p* = 0.84
D2	Kruskall-Wallis *p* = 0.27	—	—	—	—
BDNF	*F*_(3)_ = 3.04, *p* = 0.04	***p*** ** = 0.03**	*p* = 0.97	*p* = 0.2	*p* = 0.88
PCNA	*F*_(3)_ = 1.54, *p* = 0.21	—	—	—	—

### mRNA abundance

**Figure 4:**
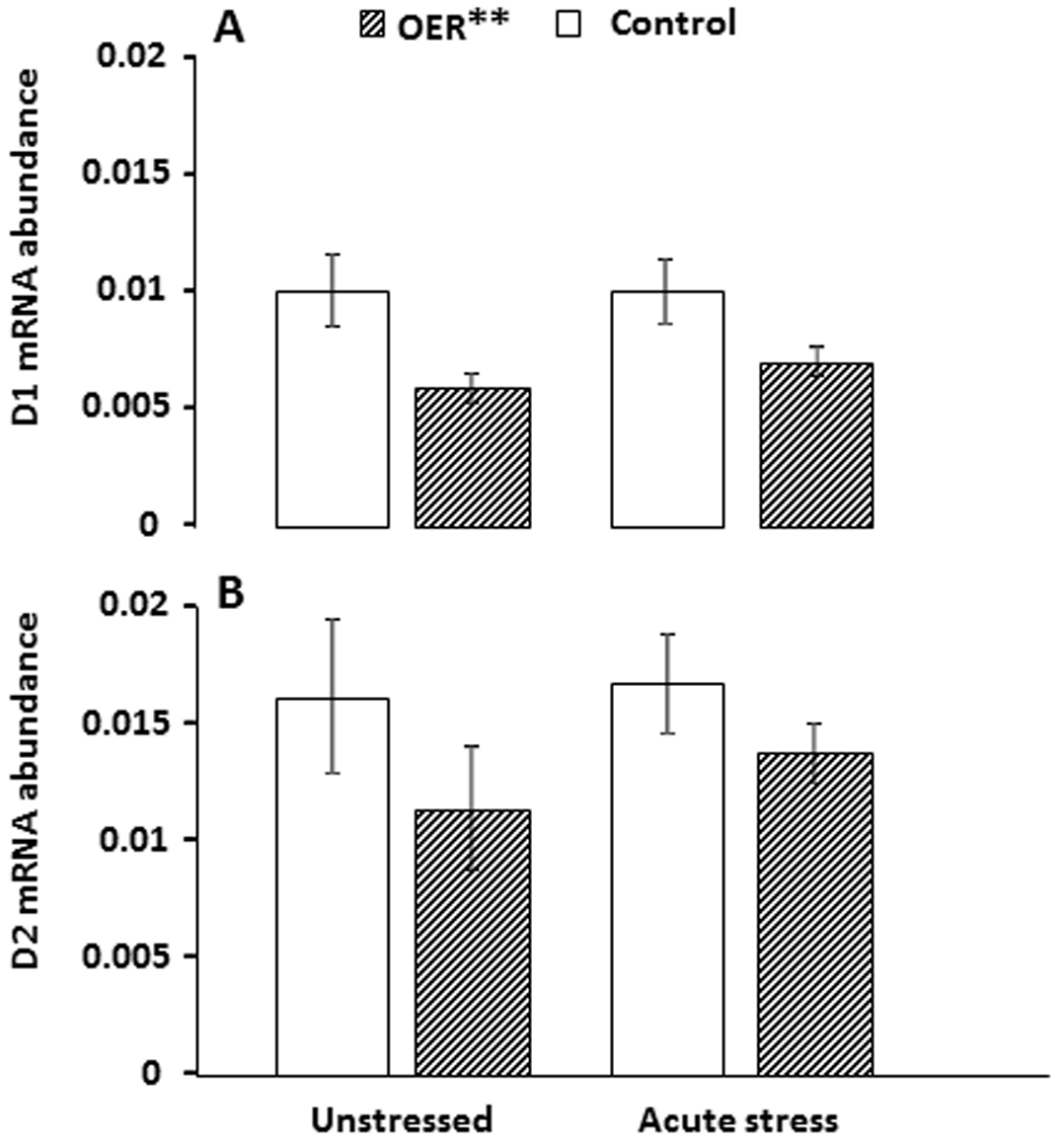
Mean (± SEM) D1 (A) and D2 (B) receptor mRNA abundance (mRNA receptor abundance normalized to EF1α mRNA abundance) in telencephalon of omission of expected reward (OER) treated and control groups under unstressed and stressed conditions. A: *ANOVA* statistics *F*_3_ = 3.14, *p* = 0.03 followed by a *Tukey HSD* post-hoc test. An additional one-way *ANOVA* was conducted to elucidate overall effects of OER treatment, *F*_(3)_ = 8.95, *p* = 0.004 (Note that OER** refers only to D1).B: *Kruskall-Wallis test*, *p* = 0.27.

**Figure 5:**
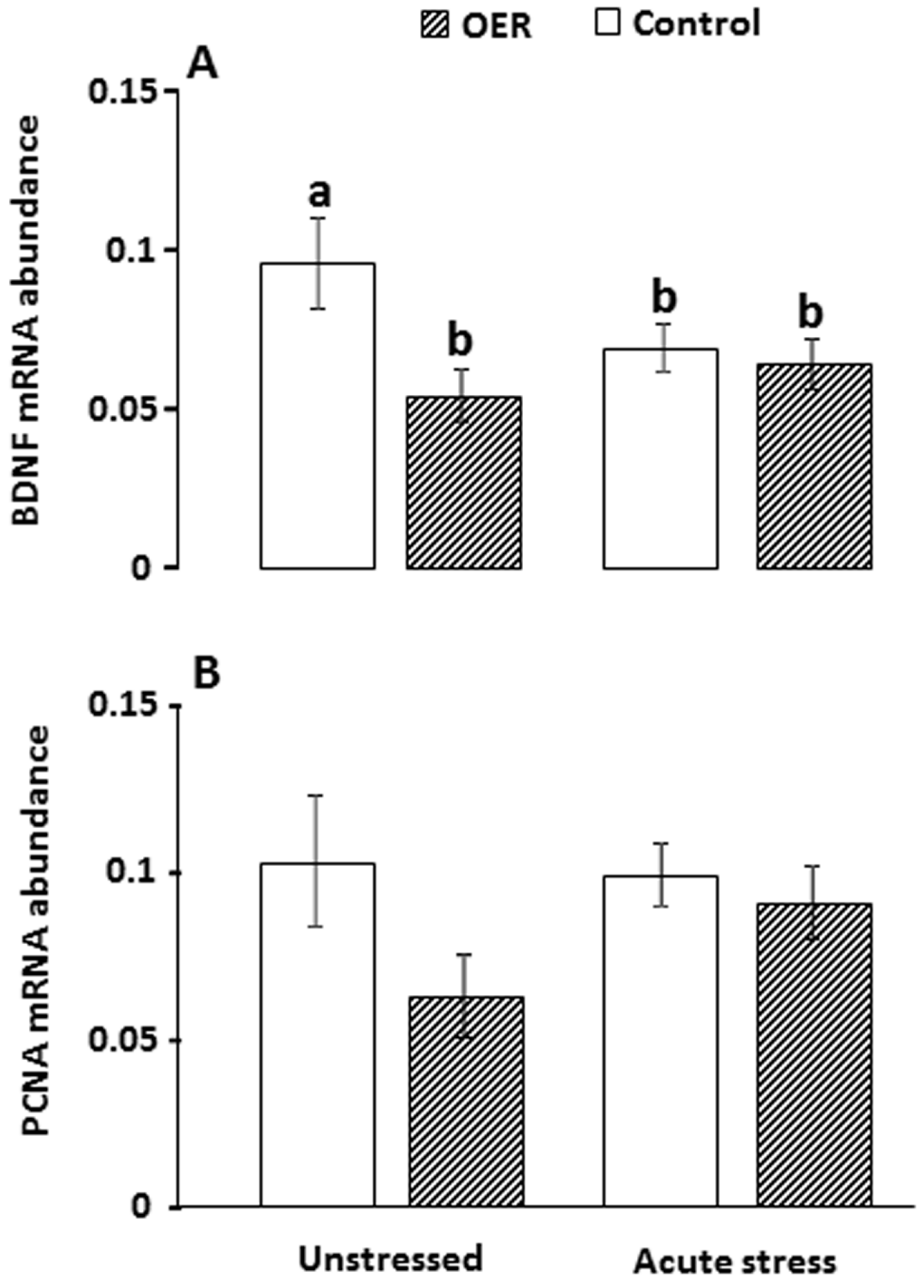
Mean (± SEM) BDNF (A) and PCNA (B) mRNA abundance (e.g. BDNF mRNA abundance normalized to EF1α mRNA abundance) in telencephalon of omission of expected reward (OER) treated and control groups under unstressed and stressed conditions. *ANOVA* statistics, A: *F*_(3)_ = 3.04, *p* = 0.04, and B: *F*_(3)_ = 1.54, *p* = 0.21. *Tukey HSD* post-hoc significance levels are indicated by differing letters when *p*<0.05.

ANOVA indicated a significant effect on relative telencephalic D1 receptor mRNA abundance (*p* = 0.03, see figure 4A caption for detailed ANOVA statistics), but post-hoc testing did not pinpoint significant differences between groups (Table 1). An additional one-way ANOVA model revealed a highly significant effect of treatment (OER vs. control, *F*_(3)_ = 8.95, *p* = 0.004). There were no group differences in D2 mRNA abundance (*p* = 0.31, Figure 4B). Relative BDNF mRNA abundance was significantly reduced in the telencephalon of OER animals compared to control animals under unstressed conditions, while after acute stress there were no group differences (Figure 5A, and Table 1). PCNA mRNA abundance did not differ significantly between groups (*p* = 0.21; Figure 5B).

### BDNF correlates

**Figure 6:**
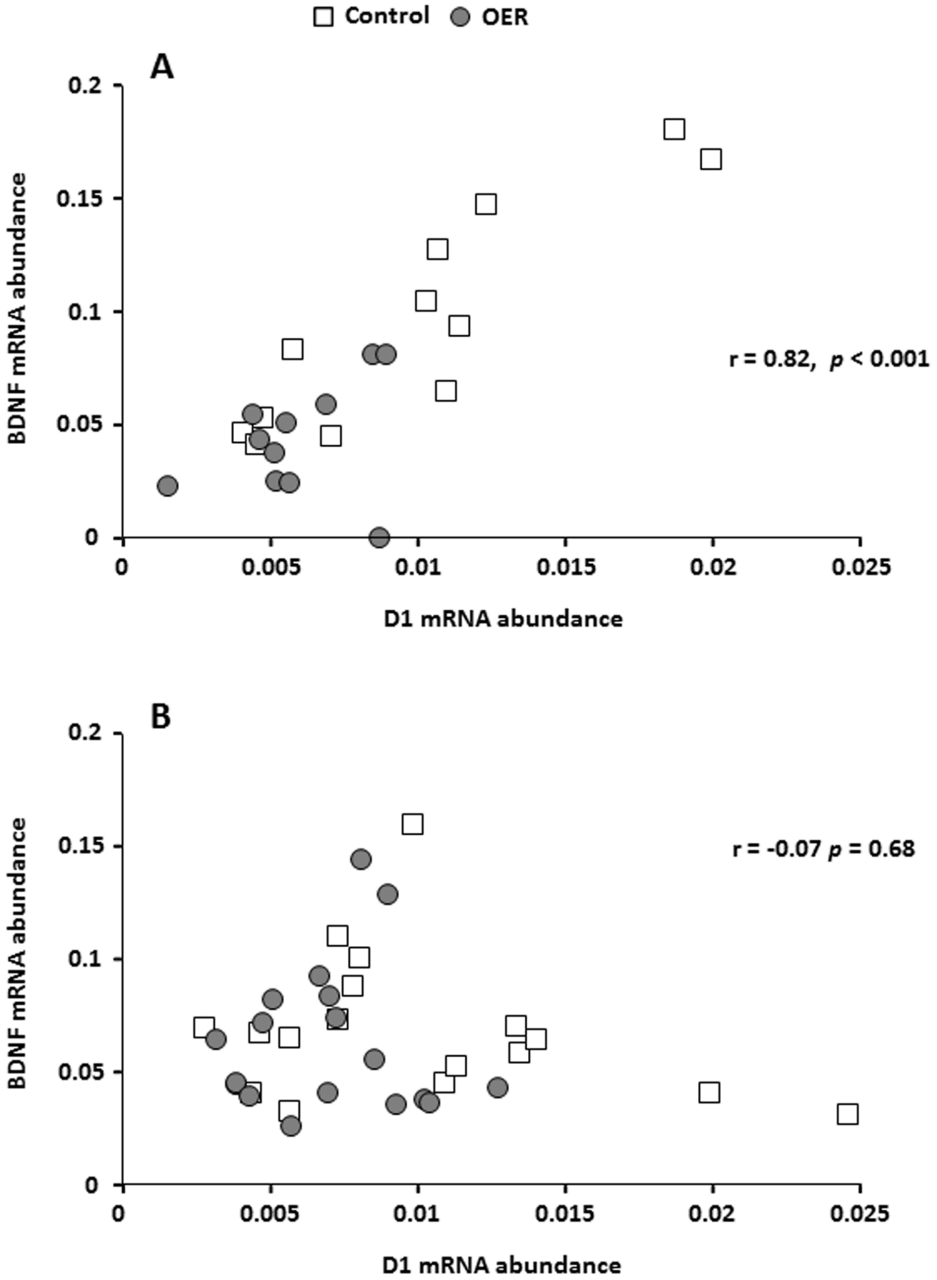
Correlation between BDNF and D1mRNA abundance for control and OER treated groups, under A) unstressed conditions (*Spearman's rho*  = 0.82, *p*<0.001), and B) after acute stress (*Spearman's rho*  = −0.07, *p* = 0.68).

**Figure 7:**
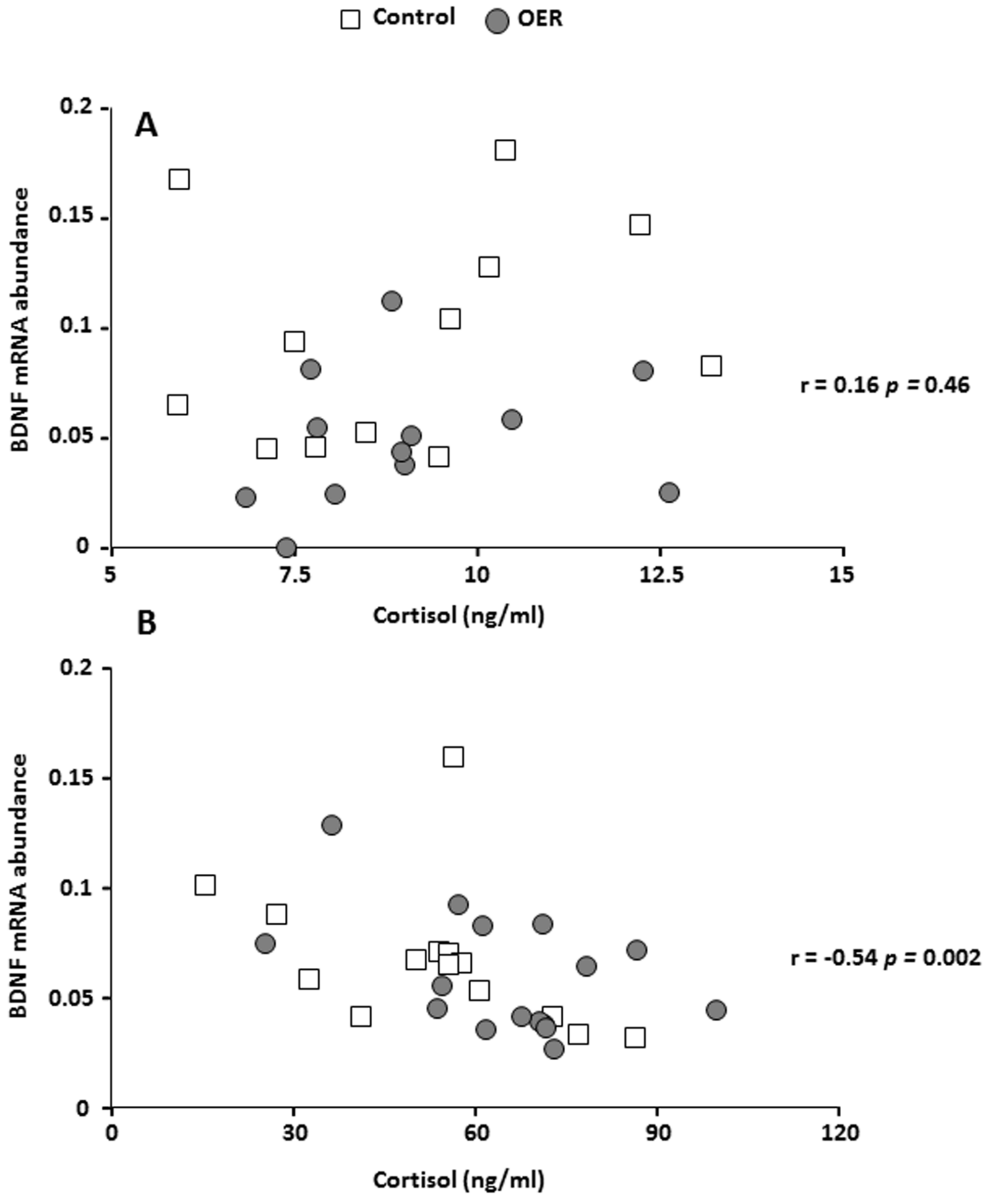
Correlation between the BDNF mRNA abundance and plasma cortisol levels of control and OER treated groups, under A) unstressed conditions (*Spearman's rho*  = 0.16, *p* = 0.46), and B) after acute stress (*Spearman's rho*  = −0.54, *p* = 0.002).

As BDNF abundance was significantly affected by OER, we analyzed pooled data for both groups using non-parametric Spearman rank correlation (ANCOVA on transformed data was used initially to verify pooling) to investigate possible correlations. There was a significant positive correlation between BDNF and D1 mRNA abundance in the telencephalon of unstressed animals (Figure 6A). In contrast, no such correlation was seen in the acutely stressed fish (Figure 6B). An opposite pattern was found for correlation between BDNF mRNA abundance and plasma cortisol levels, i.e. while there was no significant correlation in the unstressed individuals (Figure 7A), a significant negative correlation was found after acute confinement stress (Figure 7B).

## Discussion

In the current study, Atlantic salmon were trained to associate a blinking light (CS) with immediate feeding (US). When this association was well established, half of the animals were exposed to omission of expected reward (OER), which involved delaying feeding by 30 min during two out of three daily feeding sessions. This treatment caused increased aggression and growth heterogeneity, but did not affect pre- or post-stress plasma cortisol levels [19]. Presently, we show that there were also no significant differences in brain stem 5-HT or DA activity between OER and control animals under baseline conditions. Furthermore, the turnover of both monoamines was increased in control as well as in OER animals by acute stress, and the DA response was clearly potentiated in OER fish. This led us to measure forebrain mRNA abundance of DA receptors (D1 and D2). D1 mRNA abundance was found to be significantly downregulated in OER treated fish compared to controls. D2 mRNA abundance showed a similar expression pattern, but this effect did not reach statistical significance. A downregulation of brain-derived neurotrophic factor (BDNF) mRNA abundance was seen in the telencephalon of OER animals, but abundance of proliferating cell nuclear antigen (PCNA) was not significantly affected. Acute confinement stress abolished the difference in BDNF abundance between control and OER animals. Also, for both OER and control animals, BDNF abundance correlated positively with D1 mRNA abundance under baseline conditions. However, acute stress abolished this correlation. Instead, a negative correlation between plasma cortisol levels and BDNF mRNA abundance after acute stress appeared in both treatment groups.

### Structural neuroplasticity markers

In the current study, OER caused reduced mRNA abundance of BDNF, a response consistent with the hypothesis that chronic OER suppresses neuroplasticity in the telencephalon of Atlantic salmon. In mammals, brain structures related to the reward system are located in the mesocorticolimbic system of the mid- and forebrain. The ventral tegmental area in the midbrain is believed to evaluate important salient stimuli and projects to telencephalic structures. Even though the telencephalon as a whole is not homologous between fishes and mammals, recent studies have suggested homologies between different telencephalic structures, e.g. the fish's dorsolateral pallium, dorsomedial pallium and the dorsal ventral telencephalon are equivalent to the mammalian hippocampus, amygdala and nucleus accumbens, respectively. These structures located in the midbrain and telencephalon are believed to be representative of the social behavioral network, which includes the reward system in mammals and has also been proposed for teleost fishes [41], [52]–[54]. However, the reduction in telencephalic BDNF abundance may have affected structure and activity of forebrain areas associated with social behavioral networks. The difference in BDNF abundance was abolished after acute stress, suggesting an additional regulatory effect of this treatment on BDNF expression. In mammals, short-term or acute stress has in some studies been reported to stimulate hippocampal BDNF expression, whereas continued stress exposure typically leads to suppressed expression [55], [56]. Johansen et al. (2012) reported increased BDNF abundance in the optic tectum of rainbow trout (*Oncorhynchus mykiss*) in response to acute confinement stress, whereas no change in abundance was seen after long-term social stress.

Fish brains possess a very high rate of adult neurogenesis, with new neurons being formed in all major brain areas throughout the fish's lifetime. This is in contrast to the mammalian brain in which the rate of adult neurogenesis is much lower [57]. It has previously been demonstrated that environmental enrichment and acute confinement stress stimulate adult brain cell proliferation/PCNA expression, whereas chronic social stress and cortisol treatment suppress brain cell proliferation/PCNA expression in the fish telencephalon ([32]–[35], reviewed by Sørensen et al. [10]). We measured telencephalic PCNA mRNA abundance in the current study to determine whether the OER regime affected the pool of actively proliferating progenitor cells. Neither OER nor acute stress affected telencephalic PCNA mRNA abundance in the current study. This, however, does not rule out an effect of OER on adult neurogenesis in the telencephalon of Atlantic salmon, and further studies should apply immunohistochemical methods to directly investigate this possibility.

### Brain monoamine neurochemistry and DA receptor abundance

Structural neuroplasticity is modulated by monoamines in mammals [30], [31], something which may also be the case in fishes [34]. To investigate whether the change in mRNA abundance of neuroplasticity markers could relate to altered monoamine activity, 5-HT and DA activity were measured. As we were not able to analyze monoaminergic activity and gene mRNA abundance in the telencephalon simultaneously, the brain stem was used for monoamine analysis. The fish brain stem contains main nuclei of monoaminergic neurons, diffusely projecting to large parts of the brain, with strong 5-HT and DA innervation of both the dorsal (pallial) and ventral (subpallial) telencephalon [42], [43]. Although we have no direct evidence that brain stem monoaminergic activity reflects that of the telencephalon, monoamine levels released by these nuclei through local projections could potentially reflect those of the telencephalon. Monoamine activity patterns have generally been found to be coordinated between different brain areas [22], [25], [58], and brain stem 5-HT and 5-HIAA concentrations have previously been shown to correlate negatively with forebrain cell proliferation [34].

Brain stem concentrations of 5-HT and DA were not affected by neither OER nor confinement stress. There were no differences in 5-HIAA, DOPAC, 5-HIAA/5-HT, or DOPAC/DA ratios (all common measures of monoaminergic activity [22], [59]), between unstressed control and OER animals. After acute stress, markers of serotonergic and dopaminergic activity were elevated in both control and OER animals. 5-HT is involved in coordinating the neurochemical stress response in fishes and its activity consistently increases in response to stress [22], [25], an effect which can appear within minutes of stress onset [58]. DA activity, on the other hand, has in some studies been shown to increase during stress in fishes, but other studies find no such effect [60]–[62]. The lack of consistency in these results might indicate strong context-, time- and/or region-specific influence of stress on DA signaling. Most studies have also focused on social stress, and it is uncertain how this stress form compares to acute confinement stress. Nevertheless, brain stem DA activity was elevated in response to acute stress in the current study. This could suggest an information-gathering function, i.e. attention and arousal, to a novel situation, as has been reported in mammals experiencing similar situations [63], [64]. Also, DA could have a role in selection or initiation of behavior such as an escape response by promoting an increase in locomotion, which would be indicative of a coping response [64].

Notably, the DA response to acute stress was potentiated in OER animals, suggesting a sensitization of the DA system by the OER regime. DA activity is associated with the reward system in mammals, and it is believed that DA acts not only as an effective reinforcement signal activated by rewards in the learning phase [65], but that DA also facilitates the reallocation of cognitive processing capacity towards unexpected events [66]. In reward conditioning, DA activity appears to change with learning progression [21], [63], [66], [67], and certain dopaminergic neurons respond specifically to OER [21], [27]. It is thus likely, that the daily unpredictability of the feeding-related cue-reward-association encountered during OER has led to recurrent DA system activation and a sensitization of the DA system. The DA system has also been linked with aggression in fishes [24], [25], [61], [68], and we observed an increase in aggression occurring immediately after OER [19]. It is thus possible that this rapid behavioral change is induced by DA activity, although future studies should address this possibility in order to elucidate the possible role of DA in OER-related aggression.

The overall downregulation of D1 mRNA abundance in response to OER could be a compensatory mechanism reflecting the observed increase in DA responsiveness. For instance, knock-out mice (*Mus musculus*) lacking the DA reuptake transporter and therefore displaying hyperactive DA transmission have approximately 50% down-regulation of both D1 and D2 receptors [69]. Furthermore, certain DA neurons have firing patterns encoding different aspects of reward stimuli (cue-reward relationships vs. reward predictability) with potential differential effects on the D1 and D2 receptors [21]. The D1 receptor has lower affinity for DA, and is more likely to be affected by phasic DA transmission, commonly seen in DA neurons responding to cue-reward relationships and errors in reward prediction in mammals [21]. Thus, D1 receptors are potentially more likely to be affected by daily unpredictability of cue-reward-associations, as appears to be the case in the current study. A more detailed investigation should be performed in order to investigate whether the observed OER effect reflects region-specific changes in DA receptors expression.

### BDNF regulation

Abundance of BDNF mRNA was downregulated in telencephalon of OER fish. Furthermore, a significant positive correlation between forebrain BDNF and D1 mRNA abundance was found under baseline conditions. This correlation was abolished by acute stress. In mammals, D1 receptor activation stimulates BDNF expression [30], [70]–[72]. For instance, Williams et al. [30] showed that male rats (*Rattus norvegicus*) given D1 selective agonists had increased BDNF protein expression in the striatum and hippocampus. It can therefore be speculated that in the current study, BDNF downregulation results from the apparent reduction in D1 mRNA abundance, which in turn may be caused by DA hyperactivity brought on by the OER regime. It would be of interest to focus further studies on whether there is a longer-term feedback effect in operation, i.e. if reduced BDNF activity in turn serves to stabilize the DA system.

Acute confinement stress abolished the correlation between D1 and BDNF mRNA abundance, and the group difference in BDNF mRNA abundance also disappeared. A negative correlation between BDNF mRNA abundance and plasma cortisol level was evident for both control and OER animals following acute stress. This indicates an interaction between cortisol regulation and telencephalic BDNF mRNA abundance. There is ample evidence of interaction between BDNF and corticosteroid hormones within the central nervous system of mammals, although these effects can be contrasting in different brain regions and in different contexts [55], [56], [73]–[76]. Rats show a rapid increase in hippocampal BDNF mRNA in response to acute restraint stress, peaking 1 h after stress exposure. Subsequently, BDNF expression drops, and dips below baseline 2 h after stress onset [9], [74], [75]. This biphasic effect could be due to opposite effects of MR and GR glucocorticoid receptors on BDNF transcription [76]. Although there were no group-wide differences in BDNF expression after acute stress in the current study, the negative correlation with plasma cortisol indicates a possible general suppression effect of cortisol. However, since BDNF mRNA abundance is measured for the whole telencephalon, it is not possible to elucidate region-specific effects. Since there is a multitude of interaction points between BDNF and glucocorticoid regulation [55], [56], [73], further studies should address the exact relationship between the two in the fish brain.

### Conclusion

We here report that a chronic regime of OER causes DA system sensitization in the brain stem and decreased D1 receptor and BDNF mRNA abundance in the telencephalon. This indicates that both biochemical switching and structural reorganization takes place in response to OER. These changes may underlie the behavioral effect of OER (e.g. increased aggression), and may affect the animals' ability to respond to and cope with consecutive challenges. The current study was done in an aquaculture setting, demonstrating that unpredictability in rearing regimes may have unforeseen effects not only on growth and agonistic behavior [19], but also on neurobiology. It is unknown whether such changes are reversible, or if they may have long-term consequences for the animal. Nevertheless, care should be taken in optimizing animal husbandry routines to minimize unforeseen adverse effects on the animals.

As the experiment was done with large groups (n = 200 per tank), we were unable to connect neurochemistry with behavior on an individual level. Individual behavioral and neurobiological data collection should be done in order to acquire a more fine-tuned understanding of individual strategies or mechanisms under unpredictable reward conditions. Our results corroborate that fish models can be important tools in the study of neurobiological mechanisms associated with emotional responses, such as frustration. For instance, we confirm a complex, context-dependent link between DA and BDNF. The opposing roles for dopaminergic and corticosteroid BDNF regulation illustrate how neural plasticity factors respond dynamically to environmental change and may represent adaptive coping responses, providing neural mechanisms for changes in attention and perception [8]. We put forward that our work illustrates how further research should be dedicated to comparative model systems in order to understand the evolution of CNS responses to unpredictable reward conditions. In this way, fish models could serve as a new scientific approach to understanding mental processes in all vertebrates, including humans.
